# Safety assessment of petrochemical enterprise using the cloud model, PHA–LOPA and the bow-tie model

**DOI:** 10.1098/rsos.180212

**Published:** 2018-07-18

**Authors:** Qingwei Xu, Kaili Xu, Li Li, Xiwen Yao

**Affiliations:** Key Laboratory of Ministry of Education on Safe Mining of Deep Metal Mines, School of Resources and Civil Engineering, Northeastern University, Shenyang 110819, People's Republic of China

**Keywords:** safety assessment, petrochemical enterprise, cloud model, uncertainty transformation, PHA–LOPA, bow-tie model

## Abstract

Safe production is the foundation of the normal operations of petrochemical enterprises, and it helps maintain social stability. The main purpose of this study is to prevent petrochemical enterprise accidents by proposing a composite safety assessment approach based on the cloud model, preliminary hazard analysis–layer of protection analysis (PHA–LOPA) and the bow-tie model. First, the petrochemical enterprise and its relevant indicators were evaluated based on the cloud model. Second, the quantitative effect of the uncertainty transformation on the evaluation result of the cloud model was further analysed. This mainly includes the error analysis of the numerical characteristics under the conditions of few samples and small values. Third, the critical indicators such as shock and noise can be weakened and prevented by corresponding safety measures based on PHA–LOPA and the bow-tie model. After adopting two independent protection layers, the risk levels of shock and noise decrease from 3 to 2. Then, shock and noise were analysed in depth with the bow-tie model, and the causes and consequences were identified. Moreover, corresponding safety measures were taken to prevent accidents. The case study validated the validity and feasibility of the composite safety assessment approach proposed here.

## 1. Introduction

Petrochemical enterprises are the pillar industry of the national economy [1,2]. They supply the necessary resources for the rapid development of society. However, petrochemical enterprises are also a high-risk industry. Fires and explosions frequently occur [3–5], which will cause serious casualties and property loss. To prevent fires and explosions in petrochemical enterprises and build a harmonious society, first a safety assessment must be conducted.

The most frequently used quantitative safety assessment methods include the fuzzy evaluation method [6–8], grey system theory [9–11], set pair analysis [12–14], the cloud model [15–17] and neural networks [18–20]. To assess the risk of water inrush in karst tunnels, Chu *et al*. [8] proposed a two-class fuzzy comprehensive evaluation method. To solve the unquantifiable and incomplete information in the evaluation criteria, Zheng *et al*. [10] proposed a multi-hierarchical grey evaluation methodology. Tao *et al*. [14] presented a multifunctional indicator system for the performance evaluation of crop production systems using the set pair analysis method. Xu & Xu [15] introduced the synthetic cloud model to the evaluation field of ambient air quality. Taki *et al*. [20] predicted the irrigated and rain-fed wheat output energy based on an artificial neural network. In addition, game theory [21–23] was also used for evaluation. For example, Wang *et al*. [22] discussed the evolution of cooperative behaviour on two interdependent lattices in which the utility evaluation not only concern himself, but also integrate the pay-off information of several corresponding players.

Among these frequently used quantitative safety assessment methods, the cloud model was proposed by Li and co-workers [24–26]. It is a transformation between a qualitative concept described by language and its relevant quantitative value. Furthermore, the transformation is uncertain and contains fuzziness and randomness. In the safety assessment process of petrochemical enterprises, the uncertainty transformation of information will be used in many parts. Therefore, the cloud model is introduced into the safety assessment of petrochemical enterprises.

During the evaluation process of the cloud model, the uncertainty transformation has both advantages and disadvantages. Some scholars [27–29] have taken advantage of the uncertainty transformation of the cloud model to solve the practical issues. For example, to obtain the results in real time and acquire high efficiency, Wu & Zhang [27] proposed a Voronoi aesthetic pattern generation algorithm with uncertainty based on the cloud model. However, the uncertainty transformation of the cloud model will also cause errors [25]. Li *et al*. [30] studied the error caused by small initial perturbations and parameter changes in the cloud model. Nonetheless, until now, there has been little quantitative study about the effects of the uncertainty transformation on the evaluation result of the cloud model, especially for few samples and small values. Therefore, it is of great significance to study the quantitative effects of few samples and small values on the evaluation result of the cloud model, and this will be further discussed in this paper.

After evaluating petrochemical enterprises using the cloud model, the next step is taking corresponding measures to control the critical indicators. However, the cloud model cannot directly supply the control measures, and it should be combined with other safety assessment methods. Preliminary hazard analysis–layer of protection analysis (PHA–LOPA) [31–33] can identify the initiating event that leads to the accident in advance, determine the cause, risk level and consequence, and prevent the accident or reduce the risk level of the initiating event using an independent protection layer. After the critical indicators are identified by the cloud model, PHA–LOPA is suitable for reducing the risk level of critical indicators.

If there is still the possibility of an accident after using independent protection layers, further safety measures should be taken to make a detailed analysis of the critical indicators. The bow-tie model is also a widely used safety assessment method [34]. It can identify the causes that may lead to accidents and the consequences of the accident, and adopt corresponding safety measures to prevent the accident. Chen *et al*. [35] made a sample analysis of the petrochemical industry using the bow-tie method, and the influential factors of environmental risk were acquired. Pitblado & Weijand [36] described many common errors that appear in the bow tie when used for operational safety and how these might be rectified. In this paper, the bow-tie model will be used to conduct a detailed analysis of the critical indicators in petrochemical enterprises to prevent accidents.

Nonetheless, a composite safety assessment model of petrochemical enterprises is absent, and we wish to fill this gap. Therefore, the purpose of this study was to build a composite safety assessment model for petrochemical enterprises using the cloud model, PHA–LOPA, and the bow-tie model, and we regard it as an extension to previous studies of the bow-tie model [35–38]. Different from previous studies [35–38], the safety of petrochemical enterprises and its relevant indicators can be first obtained based on the cloud model. Then, the critical indicators can be weakened and prevented by corresponding safety measures based on PHA–LOPA and the bow-tie model, respectively.

This study is organized as follows. Section 2 generalizes the basic theories of the composite safety assessment model. The application of this composite safety assessment model is illustrated using a case study of petrochemical enterprises in §3. Discussions are presented in §4, and the conclusions are presented in §5.

## 2. Material and methods

The theoretical knowledge of the cloud model, the golden section method, PHA–LOPA and the bow-tie model included in this paper are presented in this section.

### 2.1. Framework of the proposed approach

**Figure 1.**
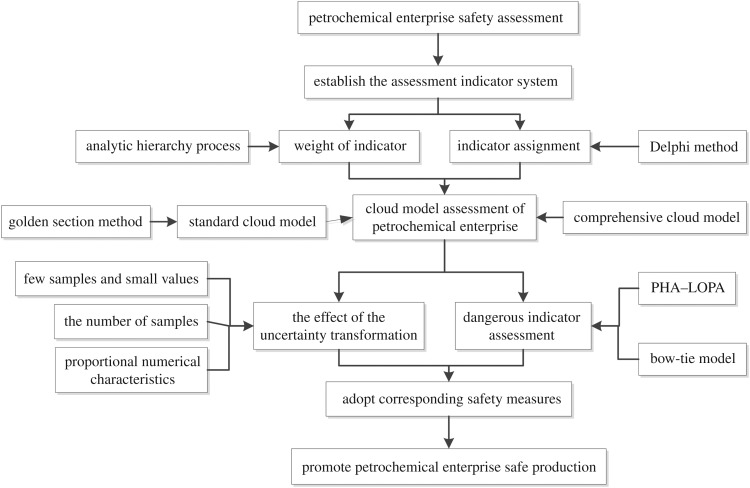
Framework of the composite safety assessment approach and the safe production of a petrochemical enterprise can be promoted by adopting corresponding safety measures.

The framework of this proposed approach is shown in figure 1.

As shown in figure 1, to assess the petrochemical enterprise, it is first necessary to establish the assessment indicator system. Second, the weight of the assessment indicator can be achieved based on the subjective weight method, such as the analytical hierarchy process [39], and assessment indicator assignment can be determined by the Delphi method [40]. Third, before the petrochemical enterprise was assessed based on the cloud model, the standard and comprehensive cloud models should be calculated in the first place. The standard cloud model is usually determined by the golden section method [41,42]. Fourth, the quantitative effect of the uncertainty transformation on the assessment result of the cloud model is further analysed, and the dangerous indicator is analysed by PHA–LOPA [31–33] and the bow-tie model [35–38]. Fifth, corresponding safety measures should be adopted to promote the safe production of the petrochemical enterprise.

### 2.2. Cloud model concept

As mentioned above, the cloud model is a transformation between a qualitative concept described by language and its relevant quantitative value. Furthermore, the transformation is uncertain, and contains fuzziness and randomness [15].

Let *U* be a quantitative domain described by a precise numerical value, and *C* be a qualitative concept in *U*. For the random element *x* ∈ *U* of the qualitative concept *C*, *μ*(*x*) ∈ [0, 1] is a random number with a stabilized trend for the membership *x* ∈ *C*. Then, the distribution of *x* in domain *U* is called the *cloud*, and *x* is called a *cloud drop*.

**Figure 2.**
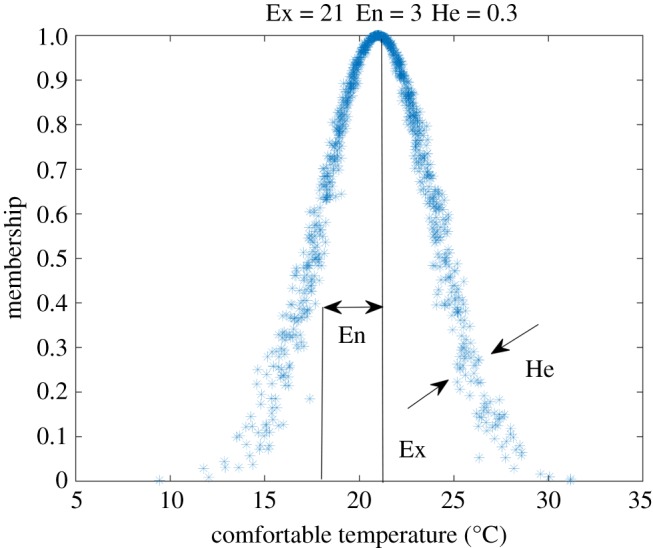
Cloud model of *comfortable temperature* with numerical characteristics (21, 3, 0.3), and this cloud image consists of 1000 cloud drops.

A specific cloud model is usually described by three numerical characteristics (Ex, En, He). A cloud model for a *comfortable temperature* is shown in figure 2, and the meanings of the three numerical characteristics are illustrated below.

The expectation Ex is the central value of the qualitative concept and the most representative of the cloud drops. The expected *comfortable temperature* is 21°C (figure 2).

The entropy En is the uncertain degree of the qualitative concept. It represents the cloud drops that are accepted by the qualitative concept in the domain. A larger En indicates greater fuzziness and randomness of the qualitative concept.

The hyper entropy He is the fuzziness and randomness of En. It represents the thickness of the cloud drops. A larger He indicates greater dispersion and randomness of the cloud drops.

### 2.3. Cloud forward algorithm

The most important algorithm of the cloud model is forward and backward cloud algorithms. The cloud forward algorithm is used to generate as many cloud drops as needed based on the three numerical characteristics (Ex, En, He). It mapped the qualitative concept to quantitative value, producing as many cloud drops as needed when the numerical characteristics (Ex, En, He) were provided. It can be easily qualitatively analysed by mapping the cloud model and standard cloud models into one cloud image. The cloud forward algorithm is neither an unambiguous membership curve nor a determinate probability density function. However, many cloud drops are created by two normal distribution functions that can realize the transformation between qualitative and quantitative. The cloud forward algorithm is described below.

*Input*. The expectation Ex, the entropy En and the hyper entropy He of the qualitative concept, and the number of cloud drops *n*.
*Output*. The position in the domain and membership of each cloud drop.
(1) Generate a normal random number En*′* with expectation En and standard deviation He.
(2) Generate a normal random number *x* with expectation Ex and standard deviation En*′*.
(3) Calculate $\mu (x)=e^{-{\left(x-\mathrm{Ex}\right)}^{2}/2{\left(\mathrm{En}\right)}^{2}}$.
(4) Repeat procedures 1–3 until *n* cloud drops are created.

### 2.4. Cloud model backward algorithm

The cloud backward algorithm is used to calculate the expectation Ex, the entropy En and the hyper entropy He from the given cloud drops. The cloud backward algorithm is described below.

*Input*: Cloud drops *x_i_* (*i* = 1,2, … ,*n*)
*Output*: Numerical characteristics (Ex, En, He) of cloud drops *x_i_*.
(1)  $\mathrm{Ex}=\frac{1}{n}\sum_{i=1}^{n}x_{i}$
(2)  $\mathrm{En}=\sqrt{\frac{\pi }{2}}\times \frac{1}{n}\sum_{i=1}^{n}|x_{i}-\mathrm{Ex}|$
(3)  $\mathrm{He}=\sqrt{\left|\frac{1}{n-1}\sum_{i=1}^{n}{\left(x_{i}-\mathrm{Ex}\right)}^{2}-{\mathrm{En}}^{2}\right|}.$

### 2.5. Comprehensive cloud model

There are complicated correlations among the safety assessment indicators, which makes it more suitable to use the comprehensive cloud model. Let the cloud model's assessment indicators be *C_i_*(Ex*_i_*, En*_i_*, He*_i_*) and the final comprehensive cloud model be *C* (Ex, En, He), in which *C_i_* is the fundamental cloud model of *C*. The comprehensive cloud model *C* can be computed as follows:

2.1 $$\begin{array}{rl} \text{Ex} & \;=\frac{\sum_{i=1}^{n}\text{E}{\text{x}}_{i}\text{E}{\text{n}}_{i}v_{i}}{\sum_{i=1}^{n}\text{E}{\text{n}}_{i}v_{i}}, \end{array}$$

2.2 $$\begin{array}{rl} \text{En} & \;=\sum_{i=1}^{n}\text{E}{\text{n}}_{i}v_{i} \end{array}$$

2.3 $$\begin{array}{rl} \text{and}\;\text{He} & \;=\frac{\sum_{i=1}^{n}\text{H}{\text{e}}_{i}\text{E}{\text{n}}_{i}v_{i}}{\sum_{i=1}^{n}\text{E}{\text{n}}_{i}v_{i}}, \end{array}$$

where *v_i_* is the weight of the assessment indicators and *n* is the number of assessment indicators.

### 2.6. Standard cloud model

The standard cloud model is a kind of cloud model which is the standard for determining the level of cloud models, and it is usually determined by the golden section method [41,42]. The main idea of the golden section method is that the closer the variable is to the domain centre, the smaller the entropy and hyper entropy of the cloud model, and vice versa. The smaller entropy of adjacent cloud models is 0.618 times the larger one; so is the hyper entropy. The standard cloud model is usually divided into an odd number of levels. It is divided into five levels in this paper, including Safe *C*_1_ (Ex_1_, En_1_, He_1_), Relatively safe *C*_2_ (Ex_2_, En_2_, He_2_), Generally safe *C*_3_ (Ex_3_, En_3_, He_3_), Relatively dangerous *C*_4_ (Ex_4_, En_4_, He_4_) and Dangerous *C*_5_ (Ex_5_, En_5_, He_5_). For the assessment indicators with two unilateral constraints [*x*_min_, *x*_max_], the numerical characteristics of the standard cloud model can be calculated as follows based on the golden section method:

2.4 $$\begin{array}{rl} \text{E}{\text{x}}_{1} & \;=x_{\max}, \end{array}$$

2.5 $$\begin{array}{rl} \text{E}{\text{x}}_{5} & \;=x_{\min}, \end{array}$$

2.6 $$\begin{array}{rl} \text{E}{\text{x}}_{3} & \;=\frac{x_{\max}+x_{\min}}{2}, \end{array}$$

2.7 $$\begin{array}{rl} \text{E}{\text{x}}_{2} & \;=\text{E}{\text{x}}_{3}+0.382\cdot \frac{x_{\max}+x_{\min}}{2}, \end{array}$$

2.8 $$\begin{array}{rl} \text{E}{\text{x}}_{4} & \;=\text{E}{\text{x}}_{3}-0.382\cdot \frac{x_{\max}+x_{\min}}{2}, \end{array}$$

2.9 $$\begin{array}{rl} \text{E}{\text{n}}_{2} & \;=\text{E}{\text{n}}_{4}=0.382\cdot \frac{x_{\max}-x_{\min}}{6}, \end{array}$$

2.10 $$\begin{array}{rl} \text{E}{\text{n}}_{3} & \;=0.618\cdot \text{E}{\text{n}}_{2}, \end{array}$$

2.11 $$\begin{array}{rl} \text{E}{\text{n}}_{1} & \;=\text{E}{\text{n}}_{5}=\frac{\text{E}{\text{n}}_{2}}{0.618}, \end{array}$$

2.12 $$\begin{array}{rl} \text{H}{\text{e}}_{2} & \;=\text{H}{\text{e}}_{4}=\frac{\text{H}{\text{e}}_{3}}{0.618} \end{array}$$

2.13 $$\begin{array}{rl} \text{and}\;\text{H}{\text{e}}_{1} & \;=\text{H}{\text{e}}_{5}=\frac{\text{H}{\text{e}}_{2}}{0.618}, \end{array}$$

where He_3_ is a constant that can be changed based on the fuzziness and randomness of the assessment indicators.

### 2.7. Similarity between the cloud model and the standard cloud model

Similarity is used to confirm the level of the cloud model evaluated, and the computational formula is as follows:

2.14 $$\lambda_{j}={\text{e}}^{-{\left(\mathrm{Ex}-{\mathrm{Ex}}_{j}\right)}^{2}/2{\left(En_{j}\right)}^{2}},$$

where Ex is the expectation of the cloud model to be evaluated, and Ex*_j_* and En*_j_* are the entropy and hyper entropy of the *j*th standard cloud model, respectively.

By computing the similarity *λ_j_* between the cloud model and the standard cloud model based on formula (2.14), the level of the standard cloud model corresponding to the maximum similarity *λ_j_* is the final evaluation result based on the maximum membership principle.

### 2.8. PHA–LOPA

Preliminary hazard analysis (PHA) is a qualitative assessment method of internal hazards and criticality. Layer of protection analysis (LOPA) is a semi-quantitative assessment method of accident scenarios that analyses the initiating event, consequences and the independent protection layer. PHA–LOPA identifies the initiating events that can lead to the accidents in advance, determine the causes, risk levels and consequences, and prevent accidents or reduce the risk level of initiating events using independent protection layers [35–38]. The risk levels of hazards are presented in table 1 according to PHA.

**Table 1.** Risk level of hazards.

level	severity	consequence
1	safe	accidents will not happen and can temporarily be ignored
2	marginal	accidents are on the threshold and may happen, which may cause casualties and property loss. Thus, countermeasures should be adopted to control the risk
3	dangerous	accidents are likely to happen, which will cause casualties and property loss. Thus, countermeasures must be adopted to control the risk
4	catastrophic	accidents will happen, which will cause serious casualties and property loss. Countermeasures must be immediately adopted to eliminate the risk

### 2.9. Bow-tie model

**Figure 3.**
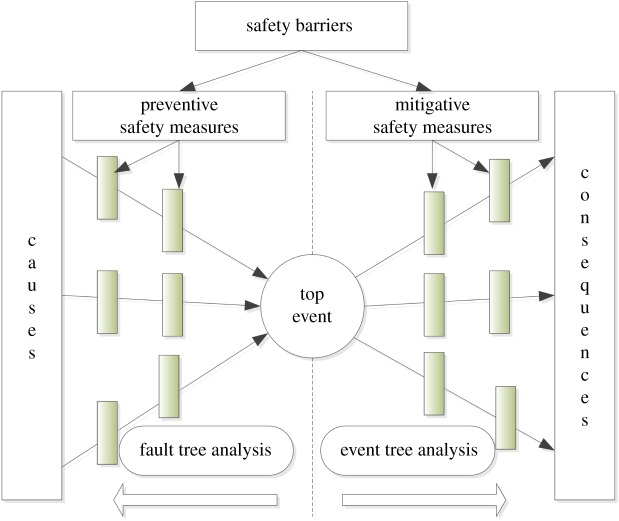
Sketch of the bow-tie model. The causes that can lead to a top event belong to the fault tree analysis, and the consequences that are caused by a top event belong to event tree analysis.

The bow-tie model (figure 3) consists of fault tree analysis on the left and event tree analysis on the right [34]. The top event is centred in the bow tie, which may happen. On the left are the basic events that may result from the top event. On the right are the consequences caused by the top event, including casualties and property loss. To prevent the top event, safety barriers must be adopted. Preventive safety measures are set on the left, and mitigative safety measures are set on the right.

## 3. Results

The production process of petrochemical enterprises is complicated and changeable, and the operation process is serialized. The producers' goods and products are flammable, combustible, poisonous, harmful and perishable. Additionally, petrochemical enterprises are prone to fire hazards and explosion accidents, which will result in serious casualties, property loss and environmental pollution. Therefore, the safety assessment of petrochemical enterprises must be conducted. It must identify the risk factors that affect safe production and propose specific improvement measures.

### 3.1. Computational process of standard cloud model

The cloud model was applied to assess the safety of a petrochemical enterprise. The safety assessment indicators are shown in table 2 [13].

**Table 2.** Information for safety assessment indicators.

			score by experts					
indicators	description	weight	1#	2#	3#	4#	5#	cloud model
*X*_1_	safety management system	0.0599	4	4	3	4	4	(3.8, 0.401, 0.198)
*X*_2_	safety organization	0.0153	5	5	5	5	4	(4.8, 0.401, 0.198)
*X*_3_	safety regulation system	0.0082	3	3	3	3	3	(3, 0, 0)
*X*_4_	contingency plan manoeuvre	0.0341	1	2	1	1	1	(1.2, 0.401, 0.198)
*X*_5_	security check	0.1427	3	3	4	3	4	(3.4, 0.602, 0.249)
*X*_6_	labour safeguard procedures	0.0423	2	2	2	2	3	(2.2, 0.401, 0.198)
*X*_7_	dangerous material	0.2539	3	3	3	3	3	(3, 0, 0)
*X*_8_	production organization	0.0280	2	2	2	3	3	(2.4, 0.602, 0.249)
*X*_9_	production characteristics	0.1330	4	5	4	4	4	(4.2, 0.401, 0.198)
*X*_10_	factory layout	0.0138	5	5	5	5	5	(5, 0, 0)
*X*_11_	lighting and illumination	0.0040	2	2	1	2	2	(1.8, 0.401, 0.198)
*X*_12_	shock and noise	0.0073	1	1	1	1	2	(1.2, 0.401, 0.198)
*X*_13_	skill and experience of staff	0.0398	3	3	3	3	4	(3.2, 0.401, 0.198)
*X*_14_	safety awareness of staff	0.0191	4	4	4	4	4	(4, 0, 0)
*X*_15_	physical condition of staff	0.0080	5	5	4	5	5	(4.8, 0.401, 0.198)
*X*_16_	mental condition of staff	0.0062	5	5	5	5	5	(5, 0, 0)
*X*_17_	safeguard system	0.0356	3	3	3	3	3	(3, 0, 0)
*X*_18_	fire-extinguishing system	0.0090	4	4	4	4	3	(3.8, 0.401, 0.198)
*X*_19_	monitoring system	0.0710	2	1	1	1	1	(1.2, 0.401, 0.198)
*X*_20_	equipment-using situation	0.0068	2	2	2	1	2	(1.8, 0.401, 0.198)
*X*_21_	equipment maintenance	0.0190	5	5	5	4	5	(4.8, 0.401, 0.198)
*X*_22_	three-level education	0.0121	3	2	3	3	3	(2.8, 0.401, 0.198)
*X*_23_	safety training	0.0220	1	1	1	1	1	(1, 0, 0)
*X*_24_	safety propaganda	0.0067	4	4	4	4	3	(3.8, 0.401, 0.198)
*X*_25_	safety activities	0.0022	5	4	5	5	5	(4.8, 0.401, 0.198)

First, each assessment indicator must be given a mark, and the standard for evaluation is presented in table 3.

**Table 3.** Standard for evaluation.

level	Safe	Relatively safe	Generally safe	Relatively dangerous	Dangerous
score	5	4	3	2	1

As shown in table 3, the standard interval for the evaluation was [*x*_min_, *x*_max_] = [1, 5]. The standard cloud model was divided into five levels according to formulae (2.4)–(2.13) of the golden section method, as given in table 4.

**Table 4.** Standard cloud model for the evaluation process.

level	standard cloud model
Safe	*C*_1_ (5, 0.413, 0.013)
Relatively safe	*C*_2_ (4.146, 0.255, 0.008)
Generally safe	*C*_3_ (3, 0.158, 0.005)
Relatively dangerous	*C*_4_ (1.854, 0.255, 0.008)
Dangerous	*C*_5_ (1, 0.413, 0.013)

### 3.2. Evaluation results of indicators

The score results of experts and weights of indicators are presented in table 2 [13]. The basic cloud model of indicators can be achieved based on the scores of experts and the backward cloud generator that are also presented in table 2.

As shown in table 2, the entropy and hyper entropy in several basic cloud models of indicators were all 0. It is because some indicators, such as *X*_1_ and *X*_7_, were scored the same by the experts, which leads to the scores of the indicators being identical to the expectation.

Taking the indicator *X*_1_ as an example, the basic cloud model of indicator *X*_1_ was (3.8, 0.401, 0.198). The similarity can be achieved based on formula (2.14). The results were *λ*_1_ = 0.015 and *λ*_2_ = 0.398, and the others were 0. Therefore, the evaluation result of indicator *X*_1_ was relatively safe based on the maximum membership principle.

The evaluation results of other indicators can be achieved in a similar way. The indicators that were evaluated Safe include *X*_2_, *X*_10_, *X*_15_, *X*_16_, *X*_21_ and *X*_25_; Relatively safe include *X*_1_, *X*_9_, *X*_14_ and *X*_18_; Generally safe include *X*_3_, *X*_5_, *X*_7_, *X*_13_, *X*_17_, *X*_22_ and *X*_24_; Relatively dangerous include *X*_6_, *X*_8_, *X*_11_ and *X*_20_; and Dangerous include *X*_4_, *X*_12_, *X*_19_ and *X*_23_.

### 3.3. Evaluation results of petrochemical enterprise

**Figure 4.**
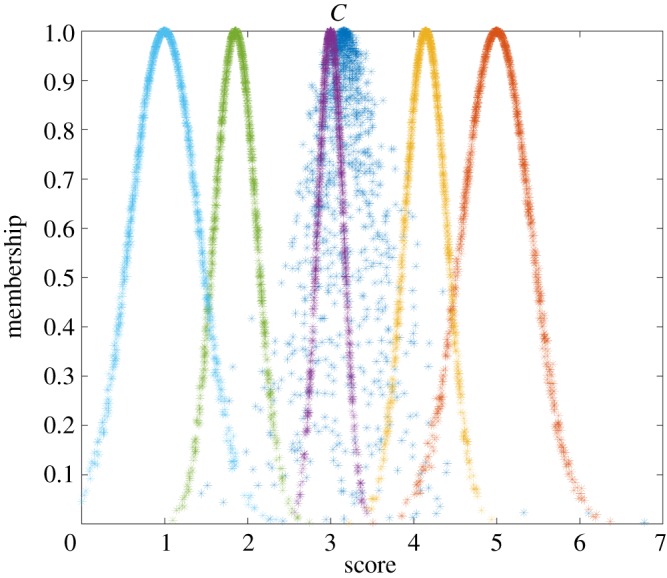
Comprehensive versus standard cloud models. The standard cloud models from left to right indicate Safe, Relatively safe, Generally safe, Relatively dangerous and Dangerous, respectively. All the cloud images consist of 1000 cloud drops.

The comprehensive cloud model of the petrochemical enterprise can be obtained according to the weights of indicators and formulae (2.1)–(2.3). The result was *C* (3.161, 0.291, 0.216). A comparison between the comprehensive and standard cloud models is shown in figure 4.

The qualitative analysis of the evaluation result can be obtained based on these images. As shown in figure 4, the comprehensive cloud model fell into the Generally safe and Relatively safe standard cloud models, which indicates that the qualitative evaluation result of petrochemical enterprises fell in between Generally safe and Relatively safe. The quantitative analysis of the evaluation result can be achieved based on similarity. The similarity can be achieved based on formula (2.14). The results were *λ*_3_ = 0.595, and others were 0. Therefore, the quantitative evaluation result of petrochemical enterprises was Generally safe based on the maximum membership principle.

Several important conclusions were reached from the process of petrochemical enterprise assessment with the present cloud model. First, an intuitive understanding and qualitative assessment were obtained by comparing the cloud model of petrochemical enterprise and its corresponding standard cloud models. Second, the greater the coverage area of the cloud model of the petrochemical enterprise, the greater is the fuzziness in determining the risk level, indicating that the score data of indicator were scattered and had violent changes in risk levels. Third, the larger the cloud thickness of the petrochemical enterprise, the greater is the randomness in determining the risk level.

## 4. Discussion

### 4.1. Quantitative effect of uncertainty transformation on the evaluation result of the cloud model

The cloud model is a transformation between a qualitative concept described by language and its relevant quantitative value. Furthermore, the transformation is uncertain and contains fuzziness and randomness. The uncertainty transformation process will inevitably cause errors. The effect of uncertainty transformation (especially for samples no larger than 30 and values no more than 5) on the evaluation result of the cloud model will be analysed in this part.

#### 4.1.1. Effect of five samples on the numerical characteristics of the cloud model

**Figure 5.**
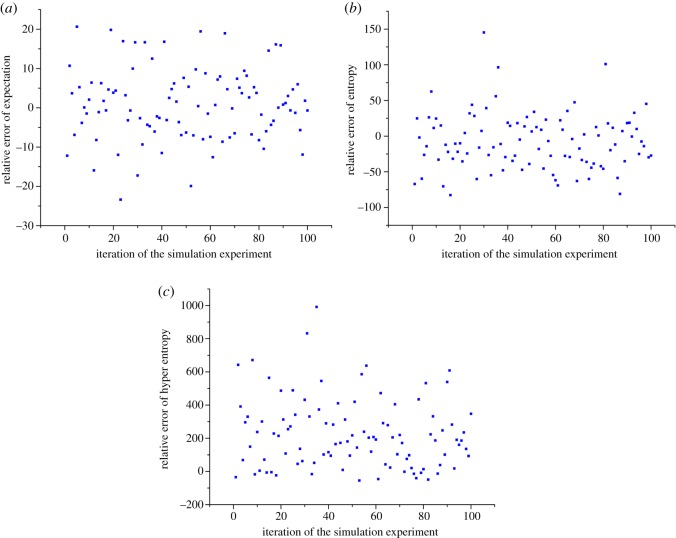
Relative error of numerical characteristics calculated by five samples with numerical characteristics (1, 0.2, 0.02), and each simulation experiment was repeated 100 times.

Let the expectation Ex* *= 1, entropy En* *= 0.2 and hyper entropy He* *= 0.02. First, generate five samples as input values with the help of the forward cloud generator. Then, calculate the numerical characteristics of the cloud model using the backward cloud generator. As the samples were randomly generated using the forward cloud generator, each simulation experiment was repeated 100 times, and finally, the relative error of the numerical characteristics was calculated. All the simulation experiments in this paper were processed with the help of the Matlab software. The relative errors of the numerical characteristics calculated by five samples are shown in figure 5.

As shown in figure 5*a*, the relative errors of expectations were approximately evenly distributed on both sides of the *y* = 0 axis, and the distribution of the sample points was denser the closer it was to the *y* = 0 axis. Most of the relative errors of the expectation fell in the interval [−20%, 20%]. The main reason is that cloud drops are created by two normal distribution functions that have a series connection, and the generating process for the cloud drops is random. Therefore, numerical characteristics of the cloud model calculated by the backward cloud generator will inevitably cause errors, and the relative error of the expectation contains randomness that considers the *y* = 0 axis as the average value.

The relative errors of entropy were also approximately evenly distributed on both sides of the *y* = 0 axis, but the range of the relative error was larger, and several relative errors of the entropy reached and exceeded 100% (figure 5*b*). This is due to the entropy being calculated by the numerical value and the expectation of the generated cloud drops. Both of them have randomness and uncertainty, which lead to further increase of the relative error of entropy.

The relative error of hyper entropy was further increased, and several relative errors of hyper entropy almost reached 1000% (figure 5*c*). This is due to hyper entropy being calculated by the entropy and the variance of generated cloud drops. Both of them have larger randomness and uncertainty than the entropy calculated by the numerical value and expectation of the generated cloud drops, which results in a larger relative error for hyper entropy.

#### 4.1.2. Effect of more samples on the numerical characteristics of cloud model

Generally, larger samples supply more information, and the numerical characteristics of the cloud model are calculated more accurately. Then, analyse the effect of more samples on the numerical characteristics of the cloud model. Additionally, let the expectation Ex* *= 1, entropy En* *= 0.2 and hyper entropy He* *= 0.02. First, generate 10, 15, 20, 25 and 30 samples as input values with the help of the forward cloud generator. Then, calculate the numerical characteristics of the cloud model using the backward cloud generator. Each simulation experiment was repeated 100 times, and finally, the relative error of the numerical characteristics was calculated. The relative errors of the numerical characteristics calculated by more samples were similar to that of the five samples, as presented in table 5.

**Table 5.** Relative errors of numerical characteristics calculated by more samples.

NS	5	10	15	20	25	30
RREEX/%	[−23.39, 20.65]	[−12.44, 15.12]	[−13.34, 12.34]	[−12.88, 9.84]	[−8.15, 12.76]	[−9.87, 9.77]
RREEN/%	[−82.71, 145.31]	[−58.94, 46.75]	[−57.80, 40.14]	[−38.96, 38.55]	[−34.86, 54.42]	[−29.19, 37.72]
RREHE/%	[−54.51, 991.01]	[−79.74, 655.92]	[−75.13, 514.99]	[−74.08, 721.51]	[−72.90, 546.56]	[−65.95, 432.20]
AAVREEX/%	7.08	4.99	4.48	3.55	3.11	2.63
AAVREEN/%	31.62	20.68	16.61	14.65	12.84	11.18
AAVREHE/%	230.67	203.38	177.19	165.22	168.48	142.92
AEHE	4.06	5.10	4.59	4.92	5.36	5.33

In table 5, NS is the number of samples, RREEX is the range of the relative error of expectations, RREEN is the range of the relative error of entropy, RREHE is the range for the relative error of hyper entropy, AAVREEX is the average absolute value of the relative error of expectations, AAVREEN is the average absolute value of the relative error of entropy, AAVREHE is the average absolute value of the relative error of hyper entropy and AEHE is the average entropy divided by the hyper entropy.

As presented in table 5, the average absolute value of the relative error of numerical characteristics gradually decreased as the number of samples increased. However, the average absolute value of the relative errors of entropy and hyper entropy was also very large, and the average for the absolute value of the relative error of hyper entropy still exceeded 100%. The entropy divided by the hyper entropy was 10 in the original cloud model, but it was approximately 5 when the numerical characteristics were calculated by the generated samples, and there was no significant change when the number of samples increased. In the future, studies should focus on how to reduce the error caused by the uncertainty transformation of the cloud model.

#### 4.1.3. Quantitative analysis between the number of samples and the relative error of expectation

As seen from formula (2.14), the most influential factor with regard to the evaluation result is the expectation among the numerical characteristics of the cloud model. The quantitative analysis between the number of samples and the relative error of expectations is carried out in this section.

**Figure 6.**
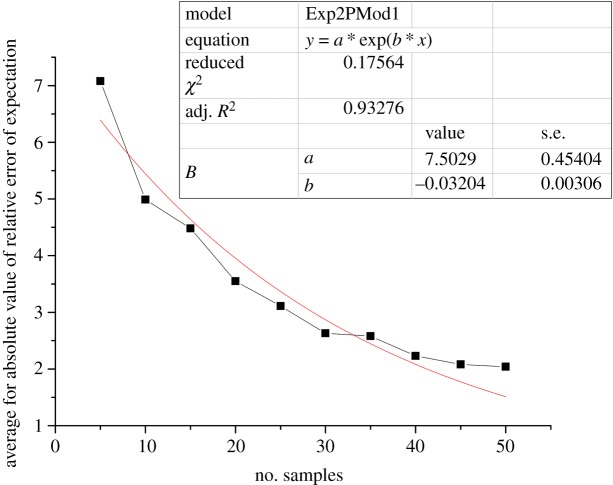
Relationship between the average absolute values of the relative error of expectations and the number of samples, and the red line is the fitting equation.

To obtain enough fitting data, let the expectation Ex* *= 1, entropy En* *= 0.2 and hyper entropy He* *= 0.02, and generate 35, 40, 45 and 50 samples as input values with the help of the forward cloud generator. Then, calculate the expectation of the cloud model using the backward cloud generator. Each simulation experiment was repeated 100 times, and finally, the relative error of expectations was calculated. The averages for the absolute value of the relative errors of expectations were 2.58%, 2.23%, 2.08% and 2.04%, respectively. The relationship between the average absolute values of the relative error of expectations and the number of samples is shown in figure 6.

As shown in figure 6, the average absolute value of the relative error of expectations gradually decreased as the number of samples increased, but it would not decrease to 0 or turn negative because the decreasing function regarded the *y* = 0 axis as the asymptotic line. Therefore, the regression formula using the exponential function is shown as follows:

4.1 $$y=7.503{\text{e}}^{-0.032x}.$$

The coefficient of determination in the above equation is large at *R*^2^ = 0.933, which shows that a reasonable fitting function was chosen.

#### 4.1.4. Error analysis for the proportional numerical characteristics

The error statistics for different samples of the same numerical characteristics were analysed above. Here, we focus on the error analysis for the proportional numerical characteristics. First, select five cloud models in which the numerical characteristics are proportional. Then, generate five samples as input values with the help of the forward cloud generator, and calculate the numerical characteristics using the backward cloud generator. Each simulation experiment was repeated 100 times, and finally, the relative error of the numerical characteristics was calculated. The error statistics for the proportional numerical characteristics are shown in table 6.

**Table 6.** Error statistics for the proportional numerical characteristics.

cloud model	(1, 0.2, 0.02)	(2, 0.4, 0.04)	(3, 0.6, 0.06)	(4, 0.8, 0.08)	(5, 1, 0.1)
RREEX/%	[−23.39, 20.65]	[−20.51, 26.57]	[−20.61, 22.35]	[−20.67, 21.70]	[−17.26, 18.59]
RREEN/%	[−82.71, 145.31]	[−80.72, 111.14]	[−70.69, 110.79]	[−60.72, 78.34]	[−84.38, 108.55]
RREHE/%	[−54.51, 991.01]	[−79.02, 861.66]	[−46.96, 783.29]	[−15.88, 822.59]	[−91.71, 755.60]
AAVREEX/%	7.08	7.21	6.42	7.06	7.32
AAVREEN/%	31.62	30.48	29.11	28.57	31.57
AAVREHE/%	230.67	246.90	212.82	241.15	231.82
AEHE	4.06	3.87	3.74	3.29	4.12

As shown in table 6, when the numerical characteristics were proportional, the relative errors of the numerical characteristics showed no significant change.

### 4.2. Effect of uncertainty transformation of the cloud model on the evaluation result

As was analysed above, large errors are caused by the uncertainty transformation of the cloud model. Take five samples as an example. The relative errors of expectations, entropy and hyper entropy could reach ±20%, 100% and even 1000%, respectively, which were all caused by the uncertainty transformation of the cloud model. It can be seen that the greatest impact on the evaluation results is the expectation of the cloud model based on formula (2.14), while the entropy and hyper entropy do not directly impact the evaluation result. In addition, the relative errors of entropy and hyper entropy were large, and it is not easy to grasp their impacts on the evaluation results. To specifically investigate the uncertainty transformation of the cloud model on the evaluation result, the hypothesis is that the entropy and hyper entropy are unchanged and just take the error of expectations caused by the uncertainty transformation into account.

**Figure 7.**
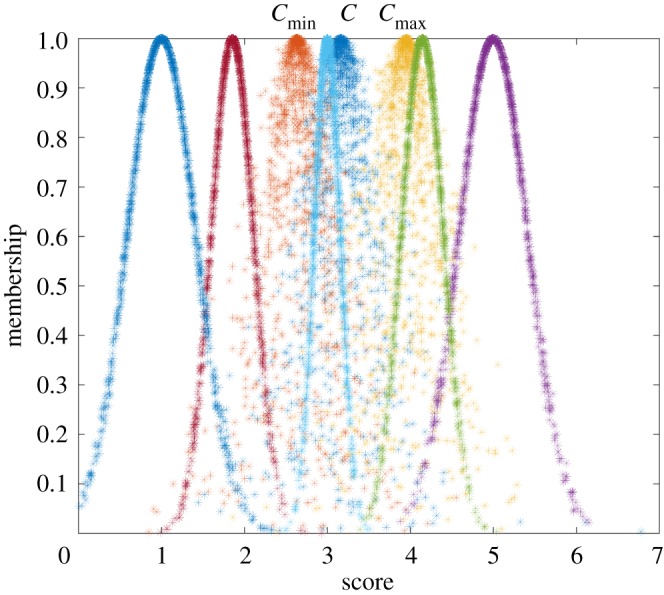
Effect of the uncertainty transformation of the cloud model on the evaluation result; the comprehensive cloud model of petrochemical enterprise may fall into different standard cloud models.

The comprehensive cloud model of this petrochemical enterprise was *C* (3.161, 0.291, 0.216), and changed the expectation on the basis of the relative error by +20% and −20%, respectively. Therefore, the worst situation of cloud model may be *C*_min_ (2.634, 0.291, 0.216), and the best situation may be *C*_max_ (3.951, 0.291, 0.216). The effect of the uncertainty transformation of the cloud model on the evaluation result is shown in figure 7.

As shown in figure 7, the worst safety situation of this petrochemical enterprise was between Relatively dangerous and Generally safe, and it is preferred to Generally safe. When the comprehensive cloud model was *C*_min_ (2.634, 0.291, 0.216), the similarity could be achieved based on formula (2.14). The results were *λ*_3_ = 0.068 and *λ*_4_ = 0.009, and the others were 0. Therefore, the worst evaluation result was Generally safe based on the maximum membership principle.

The middle safety situation was between Generally safe and Relatively safe, and it is preferred to Generally safe. The middle safety situation was Generally safe by calculating the similarity based on the maximum membership principle.

The best safety situation was between Generally safe and Relatively safe, and it is preferred to Relatively safe. When the comprehensive cloud model was *C*_max_ (3.951, 0.291, 0.216), the similarity could be achieved based on formula (2.14). The results were *λ*_1_ = 0.040 and *λ*_2_ = 0.747, and the others were 0. Therefore, the best evaluation result was Relatively safe based on the maximum membership principle.

It can be seen from the above analysis that if considering the effect of the uncertainty transformation of the cloud model, the evaluation result may be different and skip from one level to another level.

Future studies should focus on how to reduce the effects of the uncertainty transformation of the cloud model on the evaluation result or propose a forward new algorithm for uncertainty transformation.

### 4.3. PHA–LOPA of dangerous indicator

Based on the above analysis of the cloud model, the evaluation result of the petrochemical enterprise was likely to be Generally safe, and the best evaluation result was Relatively safe. Therefore, accidents were probable in the petrochemical enterprise. Moreover, the shock and noise indicator was Dangerous. Therefore, accidents in the petrochemical enterprise may be caused by the shock and noise indicator, and this indicator belongs to risk level 3 based on table 1. For this reason, the shock and noise indicator was chosen as the event scenario and analysed with PHA–LOPA, as presented in table 7.

**Table 7.** PHA–LOPA of shock and noise.

event scenario	casualties and equipment trouble caused by shock and noise
causes	(1) Mechanical unbalance; (2) Object strike
consequences	(1) Equipment trouble; (2) Casualties
risk level	3
independent protection layer	(1) Put the equipment in a right place; (2) Set safe distance
residual risk level	2
suggestion	There is still the possibility of an accident. It is recommended to continue to augment accident prevention.

### 4.4. Bow-tie analysis of shock and noise

After PHA–LOPA on the shock and noise indicator and adopting two independent protection layers, although the risk level descended from 3 to 2, it was still necessary to continue to add to the accident prevention.

**Figure 8.**
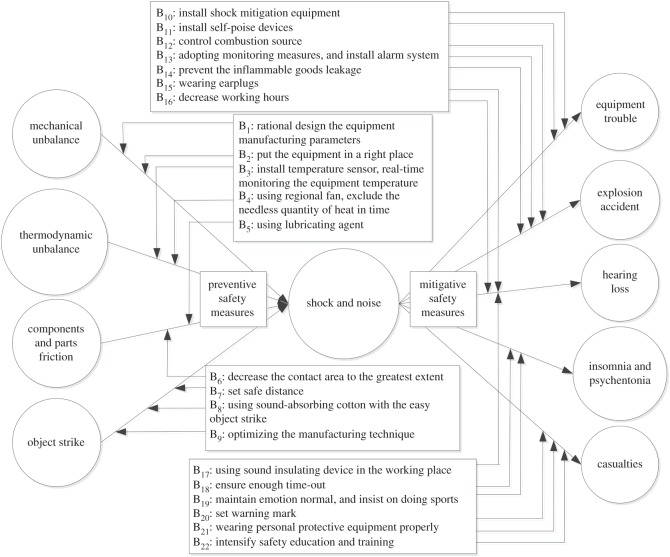
Bow-tie analysis of shock and noise; the causes and consequences were identified and corresponding safety measures were adopted to prevent accidents.

We adopted the shock and noise indicator as the event scenario, made a detailed analysis with the bow-tie model, identified the causes of the event scenario and the corresponding consequences, and adopted safety measures to prevent the event scenario, as shown in figure 8.

Four causes that can lead to shock and noise are on the left of the bow tie, which belongs to the fault tree analysis. Five consequences that are caused by shock and noise are on the right of the bow tie, which belongs to the event tree analysis. To prevent the occurrence of shock and noise, nine preventive safety measures were set on the left. To mitigate accidents’ consequences caused by shock and noise, 13 mitigative safety measures were set on the right. Therefore, the risk level of accidents' consequences caused by shock and noise can be further reduced by means of bow-tie analysis.

### 4.5. Brief summary of discussion

The present results confirmed that the composite safety assessment model proposed in this paper can be successfully applied to the evaluation of petrochemical enterprises, in which the safety of the petrochemical enterprises and its relevant indicators can be first obtained based on the cloud model. Then, the critical indicators can be weakened and prevented by corresponding safety measures based on PHA–LOPA and the bow-tie model, respectively. The advantages of the composite safety assessment model proposed in this paper were as follows. First, the safety of the petrochemical enterprise and its relevant indicators were evaluated in order to have a clear understanding of the security status and weak links of the petrochemical enterprise. Second, the effects of a few samples and small values on the evaluation result of the cloud model were analysed for the first time, and the best and worst safety situations were achieved. Third, to ensure the safe production of the petrochemical enterprise, the identified critical indicators can be weakened and prevented by corresponding safety measures based on PHA–LOPA and the bow-tie model, respectively.

Motivated by the previous studies of the cloud model [15–17], PHA–LOPA [31–33] and the bow-tie model [35–38], these three methods were used for the safety assessment of petrochemical enterprises for the first time. Different from the frequently used quantitative safety assessment methods such as the fuzzy evaluation method [6–8], grey system theory [9–11], set pair analysis [12–14] and neural networks [18–20], the cloud model uses an uncertainty transformation that contains fuzziness and randomness, which is suitable for petrochemical enterprises. Therefore, the cloud model is introduced in this field. In previous studies of the cloud model [15–17], there is little quantitative research about the effects of uncertainty transformations on the evaluation results, especially for few samples and small values, and this quantitative effect was further studied in this paper. Owing to the uncertainty transformation, relative errors of expectations fell in the interval [−20%, 20%], but entropy and hyper entropy reached 100% and 1000%, respectively. The relative error of numerical characteristics gradually decreased as the number of samples increased, but the entropy divided by the hyper entropy remained basically unchanged. When the numerical characteristics were proportional, the relative error of numerical characteristics showed no significant change. By taking the effects of the uncertainty transformation of the cloud model into consideration, the evaluation result may be different and skip from one level to another level. We imitated previous studies of PHA–LOPA [31–33] and the bow-tie model [35–38] that were applied in risk analysis. After adopting two independent protection layers, the risk level of shock and noise decreased from 3 to 2. Then, shock and noise was taken as a critical indicator for the bow-tie analysis, thus identifying the causes and consequences and taking corresponding safety measures to prevent accidents. The composite safety assessment model can be applied to the risk analysis of other related industries.

To simplify the discussion, there is not enough research on how to reduce the effects of uncertainty transformations. Future studies should focus on how to reduce the effects of uncertainty transformations in the cloud model on the evaluation results or propose a new algorithm for uncertainty transformation.

## 5. Conclusion

A composite safety assessment model of petrochemical enterprises based on the cloud model, PHA–LOPA and the bow-tie model was proposed, and the main conclusions are as follows.

First, the petrochemical enterprise and its relevant indicators were evaluated based on the cloud model. The indicators that were evaluated as Safe include *X*_2_, *X*_10_, *X*_15_, *X*_16_, *X*_21_ and *X*_25_; Relatively safe include *X*_1_, *X*_9_, *X*_14_ and *X*_18_; Generally safe include *X*_3_, *X*_5_, *X*_7_, *X*_13_, *X*_17_, *X*_22_ and *X*_24_; Relatively dangerous included *X*_6_, *X*_8_, *X*_11_ and *X*_20_; and Dangerous include *X*_4_, *X*_12_, *X*_19_ and *X*_23_. Overall, the petrochemical enterprise was evaluated as Generally safe.

Second, the quantitative effect of uncertainty transformation on the evaluation result of the cloud model was analysed. The relative errors of expectations fell in the interval [−20%, 20%], but entropy and hyper entropy reached 100% and 1000%, respectively. The relative error of numerical characteristics gradually decreased as the number of samples increased, but the value of entropy divided by hyper entropy remained almost the same. The functional relationship between the average absolute values of the relative errors of expectations and the number of samples was achieved. Then, the numerical characteristics were proportional, and the relative error of numerical characteristics showed no significant change. By taking the effects of the uncertainty transformation of the cloud model into consideration, the evaluation result may be different and skip from one level to another level.

Third, the risks of shock and noise can be weakened and prevented by corresponding safety measures based on PHA–LOPA and the bow-tie model. After adopting two independent protection layers, the risk level decreased from 3 to 2. Then, shock and noise were analysed in depth with the bow-tie model, which identified causes and consequences and led to the adoption of corresponding safety measures to prevent accidents.

## Supplementary Material

Code

## Data accessibility

The code supporting this paper has been uploaded as the electronic supplementary material.
