# Supplementary material for: Safety assessment of petrochemical enterprise using the cloud model, PHA–LOPA and the bow-tie model
Source: R Soc Open Sci. 2018 Jul 18;5(7):180212. doi: 10.1098/rsos.180212 (PMC6083725; doi:10.1098/rsos.180212)
Supplement: Code [file rsos180212supp1.doc]

***Royal Society Open Science***

Safety assessment of petrochemical enterprise using the cloud model, PHA-LOPA and the bow-tie model

**Qingwei Xu, Kaili Xu, Li Li and Xiwen Yao**

Key Laboratory of Ministry of Education on Safe Mining of Deep Metal Mines, School of Resources and Civil Engineering, Northeastern University, Shenyang 110819, People's Republic of China

Author for correspondence:

Kaili Xu

e-mail: [xklsafety@163.com](mailto:xklsafety@163.com)

**MATLAB code of cloud forward algorithm**

Ex=ex;

En=en;

He=he;

for i=1:n

% n is the number of cloud drops

Enn=randn(1)*He+En;

x(i)=randn(1)*Enn+Ex;

y(i)=exp(-(x(i)-Ex)^2/(2*Enn^2));

end

plot(x,y)

ylabel('Membership');

xlabel('Assessment score');

**MATLAB code of cloud backward algorithm**

M=[Dataset];

n=length(M);

Ex=mean(M)

En=0;

for i=1:n

En=En+(3.14/2)^(1/2)*(1/n)*abs(M(i)-Ex);

end

En

He=(abs(var(M)-En^2))^0.5
